# Repurposing Tyrosine Kinase Inhibitors for Sickle Cell Disease: Focus on Band 3 Phosphorylation

**DOI:** 10.3390/biomedicines14071500

**Published:** 2026-07-02

**Authors:** Raj Gupta, Neha Mishra, Manisha Madkaikar, Rohit Kumar Singh

**Affiliations:** 1 Indian Council of Medical Research, National Institute for Research on Blood and Immune Disorders (Formally NIIH), Mumbai 400012, MH, India; rajgupta6315@gmail.com (R.G.); madkaikarmanisha@gmail.com (M.M.); 2 Department of Pharmaceutical Sciences, Skaggs School of Pharmacy and Pharmaceutical Sciences, University of Colorado-Anschutz Medical Campus, Aurora, CO 80045, USA; neha.mishra@cuanschutz.edu

**Keywords:** sickle cell disease, Band 3, tyrosine phosphorylation, tyrosine kinase inhibitors, erythrocyte membrane

## Abstract

Sickle cell disease (SCD) is an autosomal recessive hemoglobin disorder that is mainly characterized by the presence of hemoglobin S (HbS; point mutation [Glu6Val] in the beta-globin gene). Under deoxygenated conditions, HbS polymerizes and serves as the primary trigger of oxidative stress in red blood cells (RBCs), promoting polymerization of Band 3, a major membrane scaffold protein that links the lipid bilayer to the spectrin–ankyrin cytoskeletal network. Phosphorylation at key residues within the cytosolic domain of Band 3 induces conformational changes that weaken ankyrin binding and enhance lateral mobility and clustering of Band 3. These effects are mediated through a coordinated network of erythrocyte tyrosine kinases, primarily spleen tyrosine kinase (SYK) and sarcoma (Src) family kinases, which act sequentially to modify distinct tyrosine residues. Structural features of these kinases, including tandem SH2 domains in SYK and conserved SH2–SH3–kinase domain architecture of Src family members, enable precise recognition of phosphotyrosine motifs and propagation of phosphorylation cascades. Sequence alignment and structural superimposition of SH2 domains across studied kinases demonstrate a highly conserved fold that is critical for phosphotyrosine recognition, suggesting potential overlap in substrate engagement. Therapeutically, targeting these kinases has shown considerable promise, as tyrosine kinase inhibitors (TKIs) reduce Band 3 phosphorylation, restore RBC deformability, and decrease hemolysis and vaso-occlusive interactions in vitro. Thus, in this narrative review, we focus on the regulation of Band 3 by the above-mentioned tyrosine kinases, as well as the therapeutic potential of TKIs in SCD.

## 1. Introduction

**Figure 1:**
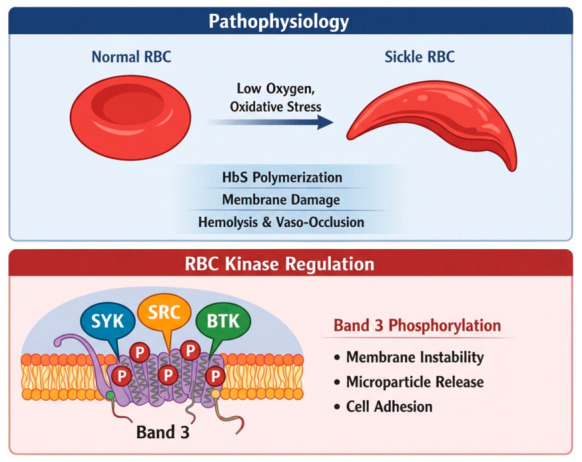
Pathophysiology of sickle red blood cells and tyrosine kinase-dependent Band 3 phosphorylation.

Sickle cell disease (SCD) is an autosomal recessive hemoglobin disorder that encompasses a group of genotypes resulting from inheritance of the sickle hemoglobin (HbS) mutation, including homozygous sickle cell anemia (HbSS) and compound heterozygous forms such as hemoglobin SC disease (HbSC) and HbS/β-thalassemia [1]. HbS results from a point mutation in the β-globin gene, where valine replaces glutamic acid at the sixth position of the β-globin chain of hemoglobin protein. This mutation gives rise to the sickle β-globin (β^S) [2], which pairs with α-globin to form the sickle hemoglobin tetramer (HbS, α_2_β_2_^S) [3]. Individuals who inherit two copies of the HbS mutation in the β-globin gene (homozygous HbSS state) develop SCD. In this review, we primarily focus on HbSS and HbSC disease in humans, as these represent the major forms relevant to the mechanisms of oxidative stress, Band 3 phosphorylation, and tyrosine kinase-mediated red blood cell-membrane dysfunction. HbS polymerizes in low-oxygen environments, causing red blood cells (RBCs) to take on the distinctive sickle shape (Figure 1). These inflexible cells impede blood flow, are prone to hemolysis, and accelerate the development of chronic anemia and vascular complications [4,5].

SCD has profoundly affected human populations for centuries. It is the most prevalent inherited blood disorder, with an estimated 300,000–400,000 affected infants born each year worldwide [6]. According to the World Health Organization, approximately 7.74 million people were living with SCD in 2021, with about 515,000 new cases reported that year [7]. Alarmingly, SCD is the 12th leading cause of death in children under five years, contributing to over 81,000 deaths in this age group in 2021. However, true mortality rates are believed to be far higher, potentially exceeding official statistics by more than ten-fold due to underdiagnosis and limited reporting [8]. As of 2025, data from the Department of Health and Family Welfare, Government of India, indicate that, in India, approximately 215,000 individuals have been diagnosed with SCD, with nearly 1,670,000 individuals identified as carriers [9].

HbS polymerization under deoxygenated conditions remains the primary initiating event in SCD, driving recurrent sickling–unsickling cycles, red cell dehydration, and oxidative stress. Beyond polymerization, another important secondary process that contributes to disease progression is the abnormal phosphorylation of Band 3, the major membrane proteins of RBCs [10]. In sickle RBCs, oxidative stress generated downstream of HbS polymerization promotes excessive tyrosine phosphorylation of the cytosolic domain of Band 3. This modification destabilizes membrane–cytoskeleton interactions, enhances vesiculation and hemolysis, and contributes to the worsening of sickle RBC dysfunction [11,12]. This excessive phosphorylation is driven by tyrosine kinases present in RBCs, particularly spleen tyrosine kinase (SYK) and Src family kinases like Lck/Yes and Lyn (Figure 1). As more studies link high Band 3 phosphorylation to membrane injury, hemolysis, and vaso-occlusion, this pathway is gaining attention as a promising target for therapy. Thus, in this narrative review, we focus on the regulation of Band 3 structure and function by these tyrosine kinases, and we explore whether existing tyrosine kinase inhibitors could be repurposed to lower Band 3 phosphorylation, stabilize the red cell membrane, and potentially improve outcomes for people living with SCD.

## 2. Role of Band 3 in SCD

**Figure 2:**
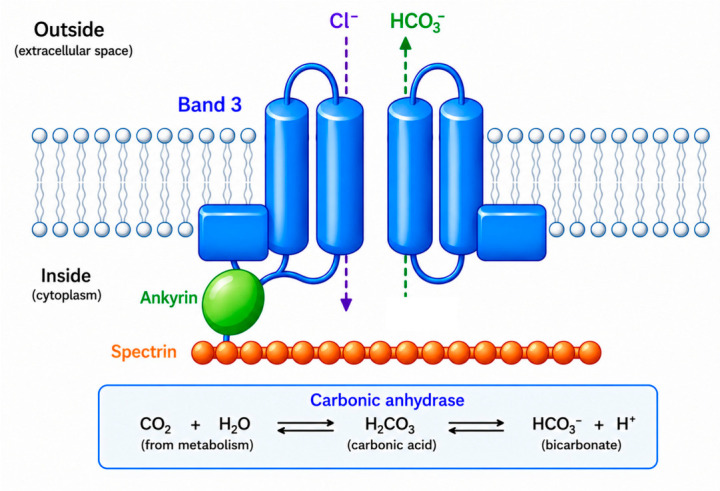
Structural organization and function of Band 3 (AE 1) in red blood cells. Band 3 consists of an N-terminal cytosolic domain (residues 1–360) that interacts with the cytoskeleton and a C-terminal transmembrane domain (residues 361–911) that mediates Cl^−^/HCO_3_^−^ exchange across the erythrocyte membrane. Adapted from Jay et al. [18].

Band 3, or anion exchanger 1 (AE 1), is the most abundant integral membrane protein present in human erythrocytes, accounting for nearly 30% of the total membrane protein content [13]. Its primary physiological role is to catalyze chloride–bicarbonate exchange across the plasma membrane, a process essential for acid–base homeostasis and efficient CO_2_ transport during respiration [14]. Structurally, Band 3 is a 911–amino acid protein (PDB: 7TW0) [15] comprising two major domains: an N-terminal cytosolic domain (residues 1–360) and a C-terminal transmembrane domain (residues 361–911) [14] (Figure 2). Band 3 has a large cytoplasmic region that connects the cell membrane to the spectrin–actin skeleton, and a transmembrane region that carries out anion exchange [16]. X-ray crystal studies of cytosolic domain of Band 3 show that it forms a dimer made of an interaction subdomain and a dimerization subdomain, which together govern its structural and regulatory functions [17].

In the context of SCD, Band 3 undergoes aberrant post-translational modifications, particularly enhanced tyrosine phosphorylation at residues Tyr 8 and Tyr 21 within the cytosolic N-terminal domain, along with oxidative modifications of cysteine residues such as Cys 201 and Cys 317 and ubiquitination, which collectively contribute to membrane instability, protein clustering, and red cell dysfunction [19,20]. Oxidative stress and recurrent sickling–unsickling cycles promote tyrosine phosphorylation of Band 3, which disrupts its interaction with ankyrin [20,21]. This destabilizes the anchoring of the spectrin–actin cytoskeleton to the lipid bilayer through the ankyrin–Band 3–protein 4.2–Rh complex [22,23], leading to loss of membrane integrity, enhanced lateral mobility of Band 3, vesiculation, and ultimately reduced erythrocyte stability [20]. These changes increase red cell clearance and contribute to both intravascular hemolysis and vascular dysfunction [20]. Quantitative analyses have demonstrated that Band 3 phosphorylation is elevated approximately 2- to 8-fold in sickle RBCs compared to healthy controls, underscoring its pathological relevance [24].

Protein tyrosine kinases, particularly SYK and Lyn, are key mediators of Band 3 phosphorylation. Sequential phosphorylation events have been reported, with SYK targeting Tyr 8 and Tyr 21, and Lyn modifying Tyr 359 and Tyr 904 [25]. This phosphorylation weakens the attachment of Band 3 to the cytoskeleton. As this connection loosens, the membrane becomes less stable and more likely to shed small vesicles. These alterations add to the stiffness and fragility of sickle RBCs and contribute towards their tendency to break apart and block small blood vessels. Importantly, oxidative stress in SCD further amplifies these effects by inactivating tyrosine phosphatases. Reactive oxygen species oxidize the catalytic cysteine residues of phosphatases such as Protein Tyrosine Phosphatase 1B (PTP1B) [26], Src Homology Region 2 Domain-Containing Phosphatase (SHP)-1 [25], and SHP-2 [27]. Loss of phosphatase activity prolongs the life time of Band 3 phosphorylation, allowing these modifications to persist rather than being rapidly reversed [28,29]. This extended phosphorylation state sustains membrane destabilization and magnifies downstream pathological effects, linking oxidative stress directly to prolonged Band 3 dysfunction in SCD. Band 3 is an evolutionarily conserved membrane protein that is widely expressed in vertebrate erythrocytes, as are the associated tyrosine kinases, suggesting that their interaction may also be evolutionarily conserved. However, the SCD-related pathophysiological features are uniquely manifested in only human RBCs. Accordingly, our study focuses specifically on the human Band 3 and human tyrosine kinases.

In SCD, inhibiting Band 3 phosphorylation has emerged as a promising therapeutic approach. Blocking SYK activity with agents such as Imatinib or more selective SYK inhibitors has been shown to reduce Band 3 phosphorylation, improve the deformability of sickle RBCs, and lower the release of microparticles and free hemoglobin [30]. These treatments also decrease the adhesion of sickle cells to the endothelium. Taken together, these observations point to Band 3 phosphorylation and its upstream kinases, particularly SYK, as meaningful targets for improving RBC stability and limiting downstream complications in SCD.

## 3. Role of RBC Tyrosine Kinases in Band 3-P and Structural Details

Erythrocytes are the most abundant cell type in the human body, with an average life span of ~120 days. To maintain homeostasis, the bone marrow produces nearly 2 million reticulocytes per second to replenish senescent cells [31]. Although mature mammalian RBCs are anucleated and devoid of organelles, they retain a functional repertoire of signaling enzymes, including several tyrosine kinases and phosphatases [32]. These enzymes, despite the minimal signaling capacity of erythrocytes, are essential for regulating cytoskeletal architecture, maintaining membrane stability, and modulating stress-responsive signaling pathways. Proteomic studies have identified a spectrum of non-receptor tyrosine kinases in RBCs, including members of the Src family (Lyn, Fyn, and Yes), as well as SYK and BTK, underscoring the importance of kinase-mediated signaling in red cell physiology and pathology [32,33,34]. Lyn and SYK are the major kinases responsible for the phosphorylation of Band 3 in erythrocytes, particularly under conditions of oxidative stress and metabolic imbalance [25].

### 3.1. SYK Family Tyrosine Kinase

**Figure 3:**
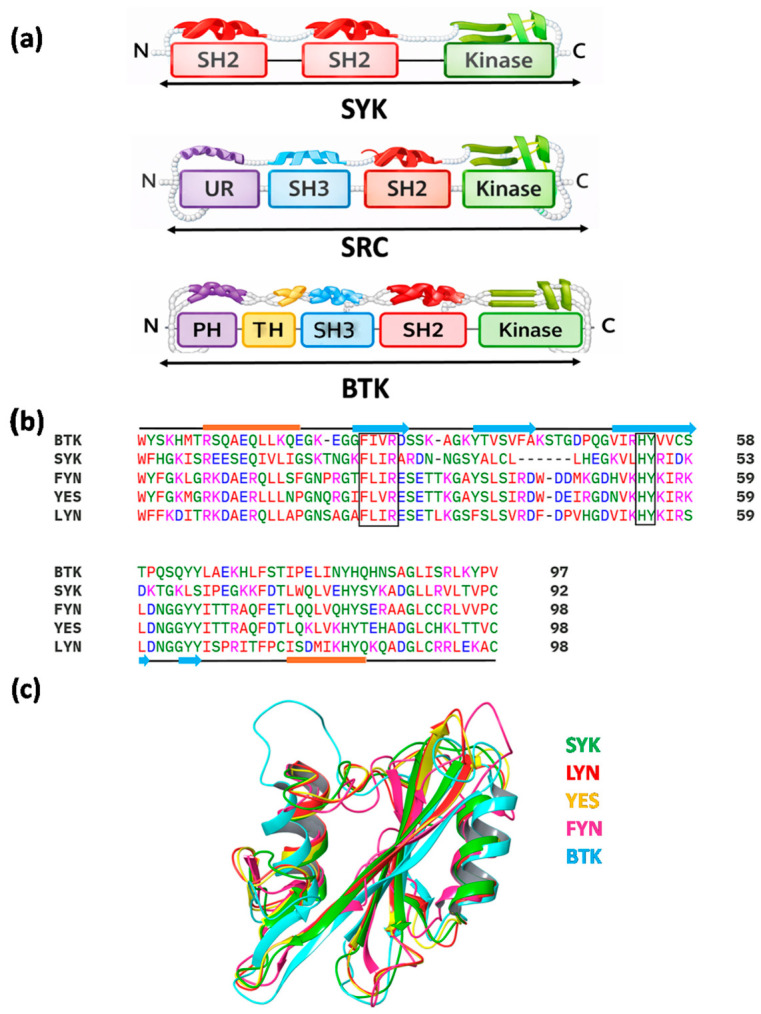
Illustration of kinase domains of interest: (**a**) Schematic domain organization of SYK, SRC, and BTK, family tyrosine kinases showing their modular architectures. (**b**) Multiple sequence alignment of the SH2 domains from representative kinases (Yes, Lyn, Fyn, SYK, and BTK), highlighting the high degree of conservation, particularly around the phosphotyrosine-binding region, including the conserved FLVR motif. Blue arrows represent α-helices and orange tubes represent β-sheets. Residues highlighted within the box denote highly conserved amino acids, including the FLVR motif, that constitute the phosphotyrosine-binding pocket of the SH2 domain and are critical for phosphotyrosine recognition across different kinase families. (**c**) Structural superimposition of SH2 domains from SYK, Lyn, Yes, Fyn, and BTK, demonstrating strong conservation of the overall SH2 fold, with near-identical three-dimensional architecture across different kinase families. SH—Src Homology domain; UR—unique region; PH—Pleckstrin Homology domain; TH—Tec Homology region.

Spleen tyrosine kinase (SYK) family kinases have a different role in RBCs compared with Src family kinases. Rather than acting as secondary signal amplifiers, SYK functions early in the signaling process and helps initiate tyrosine phosphorylation events. In erythrocytes, SYK becomes active under conditions of oxidative and mechanical stress and is responsible for the initial phosphorylation of Band 3 [29,35,36], which then leads to a wider range of changes in membrane organization. Unlike Src, SYK family kinases do not depend on lipid modifications to associate with the membrane. Instead, they are recruited through recognition of phosphorylated tyrosine residues on target proteins. This allows SYK to respond quickly to stress-induced changes at the red cell membrane, making it particularly well-suited for a cell type with limited but tightly controlled signaling capacity [35]. SYK kinases are non-receptor tyrosine kinases, structurally organized into two N-terminal SH2 domains, the N-SH2 (residues 15–107) and C-SH2 domain (residues 168–261), and a catalytic SH1/kinase domain (residues 343–621), interconnected by linker regions [37,38]. Their domain architecture consists of a tandem SH2 module separated by an inter-SH2 linker, an SH2–kinase linker, and a C-terminal kinase domain [37,38] (Figure 3a). Functionally, SYK operates immediately downstream of antigen receptors in immune cells such as B lymphocytes, mast cells, and macrophages, where it plays a central role in initiating signaling cascades [39,40,41]. Recruitment of SYK to receptors is mediated by its tandem SH2 domains, which recognize and bind to doubly phosphorylated immunoreceptor tyrosine-based activation motifs (ITAMs), canonical motifs containing two YXXL sequences separated by 7–12 amino acids [42]. Structural studies have shown that the tandem SH2 domains bind to ITAMs in a head-to-tail configuration, wherein the N-terminal pTyr-X-X-Leu of the ITAM associates with the C-terminal SH2 domain of SYK and vice versa [40]. Each SH2 domain contains a complete binding pocket capable of accommodating phosphotyrosyl residues, thereby ensuring high-affinity binding [38].

SYK activity is regulated by several phosphorylation events that shape both its catalytic output and its ability to interact with other proteins. Important regulatory sites include Tyr 348 and Tyr 352 in the SH2–linker region, which influence the shift from a closed to an open conformation [43], and Tyr 525 and Tyr 526 in the activation loop, which are required for full kinase activity and Tyr 630 in the C-terminal tail [44], which serves as a regulatory site for protein–protein interactions. Phosphorylation at these residues can occur through autophosphorylation or upon binding to ITAM peptides, both of which promote full activation of SYK. Mechanistically, SYK activation is initiated when its tandem SH2 domains bind to doubly phosphorylated ITAM motifs, relieving autoinhibitory intramolecular interactions; this conformational change facilitates SYK autophosphorylation within the activation loop, stabilizing the catalytically active kinase and enabling downstream substrate phosphorylation [45]. Activated SYK binds to phosphotyrosine motifs within the N-terminal cytoplasmic (ankyrin-binding) domain of Band 3, enabling subsequent phosphorylation of Tyr 8 and Tyr 21 [25]. These early phosphorylation events are critical, as tyrosine phosphorylation within the N-terminal cytoplasmic domain of Band 3 induces conformational and electrostatic changes that reduce its binding affinity for ankyrin. Loss of Band 3–ankyrin interaction disrupts the anchoring of the spectrin–actin cytoskeletal network to the lipid bilayer, resulting in increased membrane fluidity and reduced mechanical stability [20]. The modification of Band 3 by SYK increases its lateral mobility, promotes clustering, and creates docking sites for secondary kinases such as Lyn, which then phosphorylate additional sites on Band 3. Through this sequential activity, SYK effectively sets the stage for the broader structural changes that drive vesiculation, loss of deformability, and premature clearance of sickle red cells. Thus, the regulation of SYK and its ability to engage Band 3 lie at the heart of the phosphorylation-dependent membrane changes that contribute to sickle cell pathophysiology.

### 3.2. SRC Family Tyrosine Kinase

Sarcoma (Src) family kinases (SFKs) share a conserved modular organization that integrates membrane localization, substrate recognition, and catalytic regulation. At the N-terminus, the SH4 domain undergoes lipid modification, where myristoylation anchors the kinase to membranes, and additional palmitoylation in certain members of this family, such as Lyn and FGR, confers tight and stable membrane association. In contrast, kinases such as Yes and Fyn are only myristoylated, resulting in a more dynamic distribution between the plasma membrane and cytosol.

Following the SH4 segment, the unique region of 40–70 residues is poorly conserved and imparts isoform-specific interactions and regulatory functions. This is succeeded by two highly conserved modules: the SH3 domain (~50 residues), which binds proline-rich motifs, and the SH2 domain (~100 residues), which recognizes phosphotyrosine-containing sequences, thereby directing kinases to their phosphorylated substrates (Figure 3a). Central to SFK function is the catalytic SH1 domain/Kinase domain (~250 residues), which carries out tyrosine phosphorylation of its substrates, including Band 3 in RBCs. Finally, the short C-terminal tail harbors a conserved tyrosine residue, TYR 507 [46], whose phosphorylation maintains the kinase in an autoinhibited conformation until regulatory cues relieve this inhibition. Collectively, this hierarchical domain arrangement ensures the precise regulation of SFK localization, substrate engagement, and catalytic activity, enabling them to act as critical modulators of red cell signaling [46].

Lyn is organized into an N-terminal unique/disordered region (residues [1,2,3,4,5,6,7,8,9,10,11,12,13,14,15,16,17,18,19,20,21,22,23,24,25,26,27,28,29,30,31,32,33,34,35,36,37,38,39,40,41,42,43,44,45,46,47,48,49,50,51,52,53,54,55,56,57,58,59,60,61,62]), an SH3 domain (63–123), an SH2 domain (131–222), and a catalytic SH1/kinase domain (255–503). Lyn kinase is initially recruited to Band 3 through SH2 domain binding to phosphotyrosine residues Tyr 8 and Tyr 21 generated by SYK activity. This docking facilitates spatial positioning of Lyn on the Band 3 cytoplasmic domain, enabling subsequent secondary phosphorylation at Tyr 359 and Tyr 904 [25,47], and promoting downstream membrane remodeling and cytoskeletal dissociation.

Interestingly, sequence comparisons indicate that the SH2 domains of other Src family kinases in erythrocytes, such as Fyn and Yes, are about 80% similar to Lyn’s, whereas those of SYK and BTK (a Tec family kinase) show about 60% similarity. Despite these differences at the sequence level [25,47], structural superimposition reveals that the overall fold of the SH2 domain is highly conserved among all these kinases, with a root mean square deviation of less than 1 Å (Figure 3b,c). This SH2 domain has one highly conserved four-amino-acid-sequence FLVR motif, which is crucial for recognizing phosphorylated tyrosine residues [48,49,50,51] (Figure 3b). This motif is not only conserved in Src family kinases but also in other SH2-containing kinases, like the SYK family, ABL family, and FER/FES family, as well as members of the Tec family, including BTK. Because this FLVR motif is preserved across these several enzymes, kinases apart from Lyn may theoretically be capable of binding phosphorylated Band 3. While Lyn remains the main Src family kinase linked to secondary Band 3 phosphorylation, the shared FLVR-based recognition mechanism suggests that Fyn, Yes, and possibly other tyrosine kinases could also participate in, or influence, Band 3 phosphorylation under certain conditions.

### 3.3. TEC Family Tyrosine Kinases

Bruton’s tyrosine kinase (BTK) belongs to the tyrosine kinase expressed in the hepatocellular carcinoma (Tec) family of non-receptor tyrosine kinases. Although it is best known for its role in B-cell signaling, small amounts of BTK are also found in mature RBCs. BTK follows the typical structural layout seen in Tec family kinases. It contains a Pleckstrin Homology (PH) domain at the N-terminus, which helps the protein associate with membrane lipids. This is followed by the Tec Homology (TH) region (residues 135–210), which includes a Btk Homology region (BH domain), and then the SH3 (residues 214–274) and SH2 domains (residues 281–377) that support protein–protein interactions, as well as aid binding to phosphotyrosine-containing motifs. The C-terminal kinase domain (SH1) (residues 402–655) (Figure 3a) carries the catalytic machinery needed for phosphorylation of downstream substrates such as phospholipase Cγ2 (PLCγ2) [52].

After being recruited to the cell membrane, BTK is activated in two stages. First, SYK or Src family kinases phosphorylate BTK at position Y551 in the kinase domain [51]. This initial phosphorylation then allows BTK to auto phosphorylated at position Y223 in the SH3 domain, which increases its catalytic activity [54]. It is believed that phosphorylation at Y223 fully activates BTK kinase activity and stabilizes the active conformation [55]. The presence of the conserved FLVR motif in its SH2 domain means that BTK can recognize phosphotyrosine-containing sites, similar to SYK, Lyn, Fyn, and Yes. Since SYK is responsible for initial phosphorylation on Band 3 at Y8 and Y21, BTK could, in principle, bind to these sites through its FLVR-containing SH2 domain. Once recruited in this way, BTK may be able to further modify Band 3 or influence additional phosphorylation steps. This possibility suggests that BTK might participate in the Band 3 phosphorylation pathway indirectly by recognizing SYK-generated phosphotyrosine residues and potentially contributing to downstream phosphorylation events during red cell stress, such as those seen in SCD. However, to date, there is no direct experimental evidence demonstrating BTK recruitment to Band 3 or its role in Band 3 phosphorylation in erythrocytes. Therefore, the potential involvement of BTK should be considered hypothetical and based primarily on structural and sequence conservation rather than experimental validation mechanisms. In vitro studies can be performed to assess BTK activity and quantify phospho–Band 3 levels under oxidative stress conditions using human/patient derived sickle erythrocytes. In addition, BTK–Band 3 interaction mapping can be evaluated using co-immunoprecipitation and proximity-based proteomics.

## 4. Tyrosine Kinase Inhibitors (TKIs) and Their Potential Use in SCD

**Figure 4:**
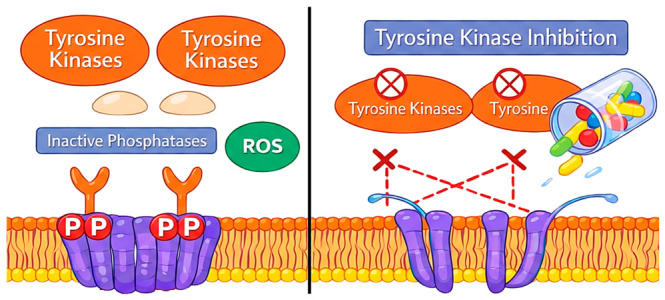
Effect of tyrosine kinase activity and its inhibition on Band 3 phosphorylation (Purple rod shape represents single Band 3 Protein). The left panel illustrates excessive tyrosine kinase activity, together with inactive phosphatases, leading to sustained Band 3 phosphorylation and clustering. The right panel shows how inhibition of tyrosine kinases reduces Band 3 phosphorylation and clustering.

As discussed earlier, sickle RBCs show excessive tyrosine phosphorylation of Band 3, and this plays a major role in RBC damage. High Band 3 phosphorylation weakens the membrane; increases microparticle shedding; releases more free hemoglobin; and contributes to hemolysis, endothelial activation, and vaso-occlusion [30]. Because these changes are driven by tyrosine kinases, blocking these kinases offers a therapeutic approach (Figure 4). One reason for increased Band 3 phosphorylation is oxidative stress in erythrocyte, which leads to reduced phosphates activity. Phosphates normally counterbalance kinase activity, maintaining a dynamic equilibrium between phosphorylation and dephosphorylation in the cell. In SCD erythrocyte, impaired phosphate function disrupts this balance, resulting in sustained Band 3 phosphorylation. In this context, TKIs may help restore the balance by reducing kinases activity and thereby modulating Band 3 phosphorylation [56] (Figure 4).

In fact, in vitro studies using RBCs from SCD patients have shown that TKIs can reduce Band 3 phosphorylation [30]. When this phosphorylation decreases, sickled RBCs regain some of their lost deformity, becoming less stiff and less likely to get trapped in small capillaries. This reduction in Band 3 phosphorylation also leads to fewer microparticles, lower free hemoglobin release, and decreased adhesion to endothelial cells [30]. Together, these findings support the idea that inhibiting the key kinases upstream of Band 3 could help stabilize sickle RBCs and improve their overall function.

Imatinib was one of the first TKIs tested in mice, as well as in humans [57,58]; some studies showed that it could partially lower Band 3 phosphorylation and improve a few sickle-related changes [30,57,58]. However, the overall effect was limited, primarily because Imatinib is a weak inhibitor of the main RBC kinases involved in Band 3 phosphorylation. Its reported IC_50_ values are about 100 µM for SYK, 100 µM for Lyn, and around 10 µM for BTK (Table 1) [59,60,61,62,63,64,65,66,67,68], much higher than levels typically needed for strong kinase inhibition. When compared with other TKIs, the gap is striking. Drugs such as Bosutinib, Dasatinib, and Ibrutinib have much stronger potency, with IC_50_ values in the nanomolar range for the same kinases [59,60,61,62,63,64,65,66,67,68]. Because they inhibit SYK, Lyn, BTK, and related enzymes far more effectively, these TKIs may offer stronger and larger number of benefits for sickle RBCs than Imatinib. Given the heterogenous nature of the available evidence, studies evaluating tyrosine kinases dependent Band 3 phosphorylation in SCD are summarized according to their level of evidence (Table 2). This classification highlights the progression from mechanistic in vitro studies and ex vivo analysis of sickle erythrocytes to preclinical animal investigations and limited clinical observation. While the collective finding support Band 3 phosphorylation as a promising therapeutic target, robust clinical evidence for tyrosine kinase inhibitor-based intervention in SCD remains limited. Despite their therapeutic potential, the translational application of TKIs in SCD requires careful consideration of long-term safety, particularly due to their systemic effects on immune function, platelet activity, and cardiovascular health. This is especially important in pediatric and young-adult SCD populations and immunocompromised patients, for whom chronic treatment may necessitate careful dose optimization and risk–benefit assessment [69,70]. Another advantage is that most of these TKIs are already approved for other diseases. This makes drug repurposing possible, which is faster than developing a new drug entirely. Overall, the evidence suggests that more potent TKIs, used alone or in combination, should be explored as potential treatments for SCD, especially since reducing Band 3 phosphorylation has benefits for RBC stability and function.

## 5. Conclusions

The growing body of evidence reviewed here highlights the central role of tyrosine kinase-driven Band 3 phosphorylation in the pathophysiology of SCD, downstream of the primary initiating event of HbS polymerization. While HbS polymerization initiates sickling, the downstream membrane damage, vesiculation, hemolysis, and loss of red cell deformability are strongly amplified by the phosphorylation-dependent disruption of the Band 3–ankyrin–cytoskeletal complex. RBC–associated kinases such as SYK, Lyn, and potentially BTK act as key regulators of this process, creating a signaling environment that promotes membrane instability under oxidative and mechanical stress. While BTK has emerged as a potential contributor, its involvement in sickle RBC signaling remains speculative and requires experimental validation. Understanding this kinase network provides a clearer mechanistic framework for the progressive membrane injury observed in sickle RBCs.

The therapeutic implications of these findings are substantial. Inhibiting the kinases responsible for Band 3 phosphorylation, particularly SYK and Src family kinases, has been shown in vitro to restore aspects of RBC stability, reduce micro particle release, and decrease cell–endothelial interactions. Although Imatinib provided the first proof-of-concept that TKIs can modulate Band 3 phosphorylation, its relatively weak activity against RBC kinases limits its usefulness. Newer TKIs with stronger potency against SYK, LYN, and BTK may offer greater therapeutic benefit and should be explored in preclinical and clinical studies. Because these agents are already approved for other indications, repurposing them for SCD provides a practical and accelerated pathway for evaluation. Despite promising in vitro and preclinical findings, several in vivo limitations must be considered for the therapeutic application of TKIs in SCD. Achieving adequate drug exposure in circulating erythrocytes may be influenced by pharmacokinetic factors such as plasma protein binding, bioavailability, and intracellular target engagement. Moreover, as most TKIs were originally developed for nucleated cells, systemic off-target effects and long-term safety remain important considerations, particularly in chronic SCD treatment. Therefore, additional in vivo pharmacokinetics and safety studies are needed to establish optimal dosing and therapeutic efficacy in SCD patients.

Taken together, the available evidence supports the view that RBC tyrosine kinase signaling is not a secondary or peripheral feature of SCD but a core contributor to red cell injury and vaso-occlusive pathology. Targeting this pathway either alone or in combination with existing SCD therapies offers a promising direction for the development of novel treatment strategies aimed at stabilizing the sickle erythrocyte and reducing disease complications. Continued investigation into the interactions among SYK, Src family kinases, BTK, and Band 3 will be essential for advancing these therapeutic opportunities. Additionally, sequence comparison of Band 3 proteins across different species revealed conservation of key tyrosine residues involved in phosphorylation (Tyr8, Tyr21, Tyr359, and Tyr904), suggesting that similar kinase-mediated regulatory mechanisms may exist across species and supporting the broader translational relevance of these pathways (Supplementary Figure S1).

## Figures and Tables

**Table 1:** Reported IC_50_ values of different tyrosine kinase inhibitors against tyrosine kinases.

Protein Kinase	IC_50_			
	Imatinib (nM)	Dasatinib (nM)	Bosutinib (nM)	Ibrutinib (nM)
SYK	10^5^	Not tested	10^3^	10^4^
LYN	10^5^	15	0.85	200
YES	Not tested	0.5	0.4	6.5
FYN	Not tested	0.2	1.8	96
BTK	10^4^	1.3	2.5	0.5

**Table 2:** Summary of available evidence supporting tyrosine kinase-mediated Band 3 phosphorylation and its therapeutic modulation in sickle cell disease. Studies are categorized by level of evidence, and only proteins with direct experimental validation are included.

Evidence Level	Experimental System	Intervention	Key Findings
In vitro	Purified proteins/kinase assays	SYK and Lyn studies	Identified sequential phosphorylation of Band 3, with SYK mediating primary phosphorylation events, and that Lyn catalyzes secondary phosphorylation at additional tyrosine residues [21,26,38].
In vitro	Human RBCs	Endogenous SYK activation	Demonstrated that oxidative stress promotes SYK-dependent Band 3 phosphorylation and membrane remodeling, establishing Band 3 as a redox-sensitive signaling hub [31,38].
Ex vivo	RBCs from SCD patients	Imatinib-mediated inhibition of Band 3 phosphorylation	Imatinib reduced Band 3 phosphorylation, improved RBC deformability, decreased microparticle release, reduced free hemoglobin release, and diminished endothelial adhesion [32].
Preclinical	Humanized SCD mice	Imatinib	Imatinib reduced sickle cell-related organ injury, vascular dysfunction, and inflammatory pathology, thus supporting therapeutic targeting of Band 3 phosphorylation pathways [60].
Clinical	SCD patients	Imatinib	Preliminary clinical evidence suggests reduction in vaso-occlusive pain crises and improvement in selected clinical outcomes with Imatinib; larger controlled studies are needed [59].

## Data Availability

No new data were created or analyzed in this study.

## Supplementary Materials

The following supporting information can be downloaded at https://www.mdpi.com/article/10.3390/biomedicines14071500/s1, Figure S1: Sequence alignment using Clustal omega tool of Band 3 protein with in different species. Conserve Tyrosine which will be Phosphorylated by the kinases are highlighted in yellow.

## Abbreviations

The following abbreviations are used in this manuscript:

SCDs	Sickle cell diseases
RBCs	Red blood cells
HbS	Sickle hemoglobin
SYK	Spleen tyrosine kinase
AE	Anion exchanger
PDB	Protein data bank
SH	Src Homology
Tyr	Tyrosine
pTyr	Phosphotyrosine
ITAM	Immunoreceptor tyrosine-based activation motif
TK	Tyrosine kinase
TKI	Tyrosine kinase inhibitor
ROS	Reactive oxygen species
BTK	Bruton’s tyrosine kinase
