# Supplementary material for: Repurposing Tyrosine Kinase Inhibitors for Sickle Cell Disease: Focus on Band 3 Phosphorylation
Source: Biomedicines. 2026 Jul 2;14(7):1500. doi: 10.3390/biomedicines14071500 (PMC13404981; doi:10.3390/biomedicines14071500)
Supplement: Supplementary file 1 [file biomedicines-14-01500-s001.zip › biomedicines-4343474-supplementary.pdf]

```
Human           MEEL----------QDDYEDMMEENLEQEEYEDPDIPESQME---------EPAAHDTEA 41
Mouse           MGDMRDHEEVLEIPDRDSEEELENIIGQIAYRDLTIPVTEMQDPEALPTE---------Q 51
Rat             MGDMQDHEKVLEIPDRDSEEELEHVIEQIAYRDLDIPVTEMQESEALPTE---------Q 51
Rabbit          MGDL----------QEDYEEVLGEILEEEEYEDPGVPMPQVEEPAASPFEPA-------- 42
Chimpanzee      MEEL----------QDDYEDMMEENLEQEEYEDPDIPESQMEEPADEPQMEEPAAHDTEA 50
Pig             MGDFQ-----------DNEERLEENLGVQDYEDQAPPTIREEDPEAQL---------TEP 40
                * ::            * *: : . :    *.*   *  . :

Human           TATDYH-------TTSHPGTHKVYVELQELVMDEKNQELRWMEAARWVQLEENLGENGAW 94
Mouse           TATDYVPSST---STPHPSSGQVYVELQELMMDQRNQELQWVEAAHWIGLEENLREDGVW 108
Rat             TATDYIPTST---STSHPSSSQVYVELQELMMDQRNQELQWVEAAHWIGLEENLREDGVW 108
Rabbit          -PTDYH--TT---TSSHPGTREVYVELQELVMDERNREPRWMEAAHWVRLEENLGQDGAW 96
Chimpanzee      TATDYR-------TTFHPGIHEVYVELQELVMDEKNQELRWMEAARWVRLEENLGENGAW 103
Pig             TATDYHTASRHLDTHKLPEQLQIGVELRELVMDEKNQEMQWMERARWVRLEENLGKDGTW 100
                  ***        :   *   :: ***:**:**::*:* :*:* *:*: ***** ::*.*

Human           GRPHLSHLTFWSLLELRRVFTKGTVLLDLQETSLAGVANQLLDRFIFEDQIRPQDREELL 154
Mouse           GRPHLSYLTFWSLLELQKVFSKGTFLLGLAETSLAGVANHLLDCFIYEDQIRPQDREELL 168
Rat             GRPHLSYLTFWSLLELQKVFSKGTFLLDLAETSLAGVANKLLDSFIYEDQIRPQDRDELL 168
Rabbit          GRPHLSYLTFWSLLELQRVFAKGTFLLDLPETSLAGVANQLLDRFIYEEQIRPQDRDELL 156
Chimpanzee      GRPHLSHLTFWSLLELRRVFTKGTVLLDLQETSLAGVANQLLDRFIFEDQIRPQDREELL 163
Pig             GRPHLSYLTFWNLLELQKAFTKGTVLLDLPEKSLPGVVVQLLDRFIFEGQIRPEHREPLL 160
                ******:****.****::.*:***.**.* *.** **. :*** **:* ****:.*: **

Human           RALLLKHSHAGELEALGGVKPAVLTRSGDPSQPLLPQHSSLETQLFCEQGDGGTEGHSPS 214
Mouse           RALLLKRSHAEDLGNLEGVKPAVLTRSGGASEPLLPHQPSLETQLYCGQAEGGSEGPSTS 228
Rat             RALLLKRSHAEDLKDLEGVKPAVLTRSGAPSEPLLPHQPSLETKLYCAQAEGGSEEPSPS 228
Rabbit          RALLLKHSHTGDLEALGGVKPVVLTRSGDPAEPLLPQQPSLETQLFCVQGEGGTEGQSPS 216
Chimpanzee      RALLLKHSHAGELEALGGVKPAVLTRSGDPSQPLLPQYSSLETQLFCEQGDGGTEGHSPS 223
Pig             RTLLLKHSHARDIEVLGEMKPALLTP-AGPSQPLLPQQPSLEVELFCEQGEGSMEGHSSE 219
                *:****:**: ::  *  :**.:**  .  ::****:  ***.:*:* *.:*. *  * .

Human           GILEKIPPDSEATLVLVGRADFLEQPVLGFVRLQEAAELE-------------AVELPVP 261
Mouse           GTL-KIPPDSETTLVLVGRANFLEKPVLGFVRLKEAVPLE-------------DLVLPEP 274
Rat             GIL-KIPPNSETTLVLVGRASFLVKPVLGFVRLKEAVPLE-------------DLVLPEP 274
Rabbit          GVLEKIPPDSEATLVLVGRATFLERPVLGFVRLQEAAMLE-------------ALELPVP 263
Chimpanzee      GILEKIPPDSEATLVLVGRANFLEQPVLGFVRLQEAAELE-------------VVELPVP 270
Pig             I-L-GKSPENLDTLVLVGQVSFLERPVLGFVRLKEPMEMEQETEQEMEQKPEETEALAVP 277
                  *    *:.  ******:. ** :********:*   :*                *  *
```

```
Human           IRFLFVLLGPEAPHIDYTQLGRAAATLMSERVFRIDAYMAQSRGELLHSLEGFLDCSLVL 321
Mouse           VGFLLVLLGPEAPHVDYTQLGRAAATLMTERVFRITASMAHNREELLRSLESFLDCSLVL 334
Rat             VSFLLVLLGPEAPHIDYTQLGRAAATLMTERVFRVTASLAQSRGELLSSLDSFLDCSLVL 334
Rabbit          VRFLLVLLGPEAPHTDYTQLGRAAATLMSERVFRTEAYLTQSRGELVHSLEGFLDCSLVL 323
Chimpanzee      IRFLFVLLGPEAPHIDYTQLGRAAATLMSERVFRIDAYMAQSRGELLHSLEGFLDCSLVL 330
Pig             LRFLMVLLGPEDPHIDYNQLGRAAATLLSEKVFHAEARVAQSKEELVHSLGGFLDCSLVL 337
                : **:****** ** **.*********::*:**:  * :::.: **: ** .********

Human           PPTDAPSEQALLSLVPVQRELLRRRYQSSPAKPDSSFYKGLDLNGGP------DDPLQQT 375
Mouse           PPTDAPSEKALLNLVPVQKELLRRRYLPSPAKPDPNLYNTLDLNGGKGGPGDEDDPLRRT 394
Rat             PPTEAPSEKALLNLVPVQKELLRKRYLPRPAKPDPNLYEAL--DGGKEGPGDEDDPLRRT 392
Rabbit          PPTDVPSEQALLSLVPVQRELLRRRYLPSPAKPDPSFYKGLDLNGGAGAPGEPEDPLQRT 383
Chimpanzee      PPTDAPSEQALLSLVPVQRELLRRRYQSSPAKPDSSFYKGLDLNGGPG-----DDPLQQT 385
Pig             PPSDTYSEKDLLSLIPVQRALLKRRKLPSPAKQEPSFYMGLDVDGGI---EDKDDPLRRT 394
                **::. **: **.*:***: **::*    *** : .:*  *  :**       :***::*

Human           GQLFGGLVRDIRRRYPYYLSDITDAFSPQVLAAVIFIYFAALSPAITFGGLLGEKTRNQM 435
Mouse           GRIFGGLIRDIRRRYPYYLSDITDALSPQVLAAVIFIYFAALSPAVTFGGLLGEKTRNLM 454
Rat             GRIFGGLIRDIRRRYPYYLSDITDALSPQVLAAVIFIYFAALSPAVTFGGLLGEKTRNLM 452
Rabbit          GRLFGGLVRDIRRRYPYYLSDITDALSPQVLAAVIFIYFAALSPAITFGGLLGEKTRNQM 443
Chimpanzee      GQLFGGLVRDIRRRYPYYLSDITDAFSPQVLAAVIFIYFAALSPAITFGGLLGEKTRNQM 445
Pig             GKLFGGLVRDIRRRYPRYLSDITDALSPQVLSAIIFIYFAALSPAITFGGLLGEKTQNLM 454
                *::****:******** ********:*****:*:***********:**********:* *

Human           GVSELLISTAVQGILFALLGAQPLLVVGFSGPLLVFEEAFFSFCETNGLEYIVGRVWIGF 495
Mouse           GVSELLISTAVQGILFALLGAQPLLVLGFSGPLLVFEEAFFSFCESNNLEYIVGRAWIGF 514
Rat             GVSELLISTAVQGILFALLGAQPLLVLGFSGPLLVFEEAFYSFCESNNLEYIVGRAWIGF 512
Rabbit          GVSELLISTAVQGILFALLGAQPLLVVGFSGPLLVFEEAFFSFCESNNLEYIVGRAWIGF 503
Chimpanzee      GVSELLISTAVQGILFALLGAQPLLVVGFSGPLLVFEEAFFSFCESNGLEYIVGRVWIGF 505
Pig             GVSELLISTSAQGIVFSLLGAQPLLVVGFSGPLLVFEEAFYSFCQSNGLEYIVGRVWIGY 514
                *********:.***:*:*********:*************:***::*.*******.***:

Human           WLILLVVLVVAFEGSFLVRFISRYTQEIFSFLISLIFIYETFSKLIKIFQDHPLQKTYNY 555
Mouse           WLILLVMLVVAFEGSFLVQYISRYTQEIFSFLISLIFIYETFSKLIKIFQDYPLQQTYA- 573
Rat             WLILLVVLVVAFEGSFLVQYISRYTQEIFSFLISLIFIYETFSKLIKIFQDYPLQESYA- 571
Rabbit          WLILLVVLVVAFEGSFLVRFISRYTQEIFSFLISLIFIYETFSKLIKIFQDHPLQGRYDH 563
Chimpanzee      WLILLVVLVVAFEGSFLVRFISRYTQEIFSFLISLIFIYETFSKLIKIFQDHPLQKTYNY 565
Pig             WLIFLVVLVVAFEGSFLVRFISRYTQEIFSFLISLIFIFETFKKLYKIFEEHPLKKDYSA 574
```

```
                ***:**:***********::*****************:***.** ***:::**:   *

Human           NVLMVPKPQGPLPNTALLSLVLMAGTFFFAMMLRKFKNSSYFPGKLRRVIGDFGVPISIL 615
Mouse           PVVMKPKPQGPVPNTALFSLVLMAGTFLLAMTLRKFKNSTYFPGKLRRVIGDFGVPISIL 633
Rat             PVVMKPKPQGPVPNTALLSLVLMVGTFLLAMMLRKFKNSTYFPGKLRRVIGDFGVPISIL 631
Rabbit          NVVMKPKPQGPLPNTALLSLVLMAGTFFFAMMLRKFKNSSYFPGMLRRVIGDFGVPISIL 623
Chimpanzee      NVLMVPKPQGPLPNTALLSLVLMAGTFFFAMMLRKFKNSSYFPGKLRRVIGDFGVPISIL 625
Pig             A-------GPSQPNTALLSLVLMAGTFFLAMLLRKFKNSSYFPGKLRRVIGDFGVPISIL 627
                            *****:*****.***::** *******:**** ***************

Human           IMVLVDFFIQDTYTQKLSVPDGFKVSNSSARGWVIHPLGLRSEFPIWMMFASALPALLVF 675
Mouse           IMVLVDSFIKGTYTQKLSVPDGLKVSNSSARGWVIHPLGLYRLFPTWMMFASVLPALLVF 693
Rat             IMVLVDTFIKNTYTQKLSVPDGLKVSNSSARGWVIHPLGLYNHFPKWMMFASVLPALLVF 691
Rabbit          IMVLVDSFIQDTYTQKLSVPSGLQVSNSSARGWVIHPLGLFSQFPIWMMFGAALPALLVF 683
Chimpanzee      IMVLVDFFIQDTYTQKLSVPDGFKVSNSSARGWVIHPLGLRSEFPIWMMFASALPALLVF 685
Pig             IMVLVDVFIEETYTQKLSVPSGFTVSNSSARGWLIHPLGQGGSFPIWMMFASVLPAMLVF 687
                ****** **: *********.*: *********:*****    ** ****.:.***:***

Human           ILIFLESQITTLIVSKPERKMVKGSGFHLDLLLVVGMGGVAALFGMPWLSATTVRSVTHA 735
Mouse           ILIFLESQITTLIVSKPERKMIKGSGFHLDLLLVVGMGGVAALFGMPWLSATTVRSVTHA 753
Rat             ILIFLESQITTLIVSKPERKMIKGSGFHLDLLLVVGMGGVAALFGMPWLSATTVRSVTHA 751
Rabbit          ILIFLESQITTLIVSKPERKMIKGSGFHLDLLLVVGMGGVAALFGMPWLSATTVRSVTHA 743
Chimpanzee      ILIFLESQITTLIVSKPERKMVKGSGFHLDLLLVVGMGGVAALFGMPWLSATTVRSVTHA 745
Pig             ILIFLETQITTLIVSKPERKMVKGSGFHLDLLLIMGMGGVAAIFGLPWLSATTVRSVTHA 747
                ******:**************:***********::*******:**:**************

Human           NALTVMGKASTPGAAAQIQEVKEQRISGLLVAVLVGLSILMEPILSRIPLAVLFGIFLYM 795
Mouse           NALTVMGKASGPGAAAQIQEVKEQRISGLLVSVLVGLSILMEPILSRIPLAVLFGIFLYM 813
Rat             NALTVMGKASGPGAAAQIQEVKEQRISGLLVSVLVGLSILMEPILSRIPLAVLFGIFLYM 811
Rabbit          NALTVMGKASSPGAAAQIQEVKEQRISGLLVSVLVGLSILMEPILSRIPLAVLFGIFLYM 803
Chimpanzee      NALTVMGKASTPGAAAQIQEVKEQRISGLLVAVLVGLSILMEPILSRIPLAVLFGIFLYM 805
Pig             NALTVMSKSSIPGAASQIQEVKEQRISGLLVAVLVGLSILMGPILSHIPLAVLFGIFLYM 807
                ******.*:* ****:***************:********* ****:*************

Human           GVTSLSGIQLFDRILLLFKPPKYHPDVPYVKRVKTWRMHLFTGIQIICLAVLWVVKSTP- 854
Mouse           GVTSLSGIQLFDRILLLFKPPKYHPDVPFVKRVKTWRMHLFTGIQIICLAVLWVVKSTP- 872
Rat             GITSLSGIQLFDRILLLFKPPKYHPDVPFVKRVKTWRMHLFTGIQIICLAVLWVVKSTP- 870
Rabbit          GVTSLSGIQLFDRILLLLKPPKYHPDVPFVKRVKTWRMHLFTGIQIICLVALWVVKSTP- 862
Chimpanzee      GVTSLSGIQLFDRILLLFKPPKYHPDVPYVKRVKTWRMHLFTGIQIICLAVLWVVKSTP- 864
```

```
Pig             GVTSLSGIQLFDRILLLFKPSKYHPDIPYAKRVRTWRMHLYTLTQIICLVVLWTVKFFPS 867
                *:***************:** *****:*:.***:******:*  *****..**.**  *

Human           ASLALPFVLILTVPLRRVLLPLIFRNVELQCLDADDAKATFDEEEGRDEYDEVAMPV    911
Mouse           ASLALPFVLILTVPLRRLILPLIFRELELQCLDGDDAKVTFDEENGLDEYDEVPMPV    929
Rat             ASLALPFVLILTVPLRRLLLPLIFRELELQCLDGDDAKVTFDEAEGLDEYDEVPMPV    927
Rabbit          ASLALPFVLILTVPLRRLLLPYIFSNLELQCLDADDAKATFDEEEGRDEYDEVTMPV    919
Chimpanzee      ASLALPFVLILTVPLRRILLPLIFRNLELQCLDADDAKATFDEEEGRDEYDEVAMPV    921
Pig             TSLALPFVLILTVPLRLFLLPLIFRKLELQCLDADDAKPNLDEEHGRDEYDEVHMPV    924
                :*************** .:** ** ::******.**** .:** .* ****** ***
```

Supplementary Figure S1: Sequence alignment using Clustal omega tool of Band 3 protein with in different species. Conserve Tyrosine which will be Phosphorylated by the kinases are highlighted in yellow.
